# Taxonomic redescription and biological notes on *Diaugia angusta* (Diptera, Tachinidae): parasitoid of the palm boring weevils *Metamasius ensirostris* and *M. hemipterus* (Coleoptera, Dryophthoridae)

**DOI:** 10.3897/zookeys.84.756

**Published:** 2011-03-01

**Authors:** Silvio Shigueo Nihei, Ronaldo Pavarini

**Affiliations:** 1 Departamento de Zoologia, Instituto de Biociências, Universidade de São Paulo, Rua do Matão, Travessa 14, n. 101, 05508-900, Cidade Universitária, São Paulo, SP, Brazil; 2 Departamento de Agronomia, Campus Experimental de Registro, Universidade Estadual Paulista, Rua Nelson Brihi Badur, n. 430, 11900-000, Registro, SP, Brazil

**Keywords:** Dexiinae, taxonomy, host record, biological control, palm, bromeliad, Brazil, Neotropical Region

## Abstract

Diaugia angusta Perty, 1833 is a Neotropical species of Tachinidae (Diptera) reported here as a parasitoid of Metamasius ensirostris (Germar, 1824) and Metamasius hemipterus (Linnaeus, 1758) (Coleoptera: Dryophthoridae) in Brazil. Several species of Dryophthoridae and Curculionidae cause damage to bromeliad and palm species, and most are regarded as pests. In the present study, the male and female of Diaugia angusta are morphologically characterized and illustrated to provide a means for the identification of this parasitoid. Data obtained from preliminary field research show that natural parasitism of Metamasius pupae by Diaugia angusta varies by year but can reach nearly 30%. A network of parasitoid-host interactions among tachinid parasitoids and coleopteran hosts reported as bromeliad and palm pests (Dryophthoridae and Curculionidae) in the Americas indicates that the species of the tribe Dexiini *sensu lato* (including Diaugia angusta) might be promising as biological control agents of these pests.

## Introduction

The Neotropical genus Diaugia (Tachinidae: Dexiinae: Dexiini) was described by Perty (1833) to include a single new species, Diaugia angusta. This species was described based on material collected from state of Minas Gerais in the southeastern Brazil. Since the description, the genus and the species appeared in the literature only through brief citations and catalogues, without any detailed study (Townsend 1939, Guimarães 1971, Tschorsnig 1985). The most relevant studies were made by Charles H. Townsend, including Diaugia in a key to Zeliini genera (Townsend 1936) and providing a morphological diagnosis of the genus (Townsend 1939). In the end of his diagnosis, Townsend (1939: 78) wrote: “Ranges in two species from Minas Geraes [sic] to Rio de Janeiro”. Apart from the type-species (Diaugia angusta), from Minas Gerais, Townsend (1939) had therefore regarded the existence of a second undescribed species, from Rio de Janeiro. The material examined by Townsend was found deposited at the Museu Nacional (Rio de Janeiro) and a detailed examination confirmed it to be conspecific with Diaugia angusta.

Metamasius Horn (Coleoptera: Dryophthoridae) is a Neotropical weevil genus comprising about 110 species, mostly associated damaging bromeliads, sugarcane, bananas and palms (Vaurie 1966, Frank 1999). Several species are considered important crop pests. Metamasius ensirostris (Germar) is known to cause damages to bananas, sugarcane and palms (Vaurie 1966, Silva 1968). Furthermore, within the attacked palms, there are reports to several species explored for “palmito” (heart-of-palm) extraction in Brazil, as Euterpe edulis Mart. (“juçara”), Euterpe oleracea Mart. (“açaí”) and Bactris gasipaes Kunth (“pupunha”, peach-palm) (Zorzenon et al. 2000). Metamasius hemipterus (Linnaeus) is an important stem-borer of sugarcane, banana, bromeliads and palms in Central and South Americas, but also introduced to other regions of the world (Vaurie 1966). Metamasius callizona (Chevrolat) is associated with bromeliads and bananas in Central America but, after introduction, it has damaged severely the native bromeliad species in tropical areas of North America (Frank and Cave 2005).

Recently, great efforts have been applied in order to find ways to control the bromeliad and palm weevils, such as the field exploration for parasitoids (Cave et al. 2003), experiments evaluating efficiency of parasitoids (Moura et al. 2006), use of pheromone-based traps (Oehlschlager et al. 1993), among others.

In the present study, comprehensive material of Diaugia from several museums was studied in detail. Within the material, there was one specimen reared on Metamasius ensirostris from the state of Santa Catarina (southern Brazil), and several specimens reared on Metamasius hemipterus from the state of São Paulo (southeastern Brazil). Additionally, there were some specimens from São Paulo reared from an undetermined Metamasius.

This paper aims to provide taxonomic support for the identification of this parasitoid and for its potential use as a biological control agent of Metamasius species. We provide a detailed morphological characterization of Diaugia angusta Perty. Both male and female specimens are characterized, the male terminalia are described and illustrated for the first time, and photographs and distribution map are presented. In addition, based on preliminary field research performed in the state of São Paulo (Brazil), some biological data on the interaction between Diaugia and Metamasius is presented. Finally, a parasitoid-host network is elaborated showing the interactions between these parasitoid flies (Tachinidae) and coleopteran hosts reported as bromeliad and palm pests (Dryophthoridae and Curculionidae) in the Americas.

## Material and methods

The examined material is deposited at the following institutions: Museu Nacional, Universidade Federal do Rio de Janeiro, Rio de Janeiro, Brazil (MNRJ); and Museu de Zoologia, Universidade de São Paulo, São Paulo, Brazil (MZSP). The morphological terminology follows mainly McAlpine (1981), Wood (1987) and Stuckenberg (1999).

The subfamily and tribal classification of Tachinidae followed here is that used in the latest Nearctic Catalogue by O’Hara and Wood (2004), which basically followed the classification scheme of Herting (1984) and Tschorsnig (1985). One example is Dexiini, into which some tribes were included under its name (*e.g.*, Zeliini, Prosenini, Theresiini as junior synonyms). Even though, the earlier classification of Neotropical Tachinidae (*sensu* Guimarães 1971) was eventually mentioned during the discussion. In this case, the old names were cited in the following way: “Zeliini”, “Theresiini”.

To elaborate the network of parasitoid-host interactions, we included all the species of Tachinidae recorded as parasitoids of species of Dryophthoridae and Curculionidae which are reported in the literature as pests of bromeliads and palms in the Americas. However, the network is complete only in that all the hosts of “Zeliini” and all the parasitoids of Dryophthoridae and Curculionidae are presented. For the other taxa in the network, neither all parasitoids nor all hosts are presented. For complementary information about them, please refer to Guimarães (1977, South America) and Arnaud (1978, North and Central Americas). Although out-of-date, these host-parasitoid catalogues still remain as comprehensive and reliable references for the Tachinidae of the Americas for the time coverage.

## Systematics

### Diaugia

Perty, 1833

Diaugia Perty 1833: 187, type species: Diaugia angusta Perty, 1833 (by monotypy).
Diaugia ; Townsend 1936: 30 (key to genera of Zeliini), Townsend 1939: 77 (diagnosis), Guimarães 1971: 101 (as ‘Diaughia’, catalogue, in Zeliini), Tschorsnig 1985: 100 (as ‘Diaughia’, citation, male terminalia characterization, in Dexiini).
Diaughia , error.

#### Diagnosis.

Diaugia differs from other South American Dexiini by the following combination of characters: eye bare; arista densely long plumose; no facial carina; parafacial bare; proepisternum bare; intrapostalar seta absent; 2 katepisternal setae; katepimeron (barette) setulose anteriorly; costal spine undeveloped; R1 without setulae; base of R4+5 setulose dorsally and ventrally; abdominal syntergite 1+2 and tergite 3 without median marginal setae (but females have a pair of median marginal setae on tergite 3) and all tergites without discal setae (but some ground setulae rather developed middorsally); male abdomen conspicuously elongate (although not caudate as in Uramya Robineau-Desvoidy and Trichodura Macquart).

Among Dexiini, Diaugia undoubtedly resembles the other genera formerly included in the extinct tribe Zeliini (sensu Guimarães 1971, 1975). If valid today, this tribe would include 11 genera, all them monotypic except for Zelia Robineau-Desvoidy with nine species. Although Diaugia may be distinguished from other “Zeliini” and Dexiini (former paragraph), as well as each of the former “Zeliini” genera may bear a set of diagnostic characters, a discussion about the validity of each of these 10 monotypic genera is extremely necessary. On the other hand, to achieve a reliable treatment of these generic names (if valid or not), a detailed and comprehensive revision including types and non-types of all the 11 genera is much required.

#### Diaugia angusta

Perty, 1833

**Figures 1–4.**
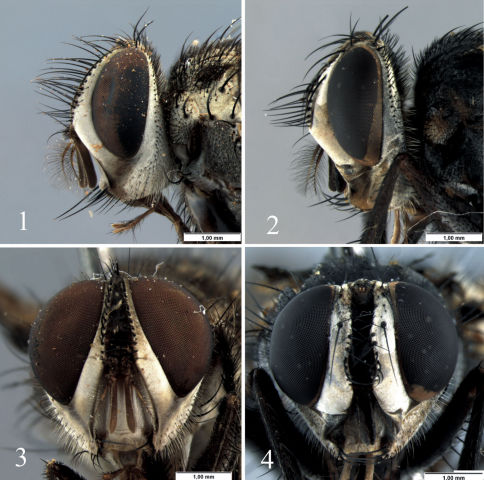
Diaugia angusta: **1** male head, lateral view **2** female head, lateral view (head partially collapsed) **3** male head, frontal view **4** female head, frontal view. (Scale bar: 1 mm)

Figures 1 [Fig F2] [Fig F3] 11

Diaugia angusta Perty 1833: 187, plate 37, fig. 9, type locality: Brazil, Minas Gerais, syntype male (lost *sec*Reiss and Schacht 1983: 308, formerly at the Zoologischen Staatssammlung München), syntype male (USNM, #A16608, also from Minas Gerais, not examined)

##### Redescription.

###### Male:

####### Body length:

13.5 mm (11.5–15.0), wing length: 11.0 mm (10.0–12.0) (n=20).

####### Colouration

**Figures 5–7. F2:**
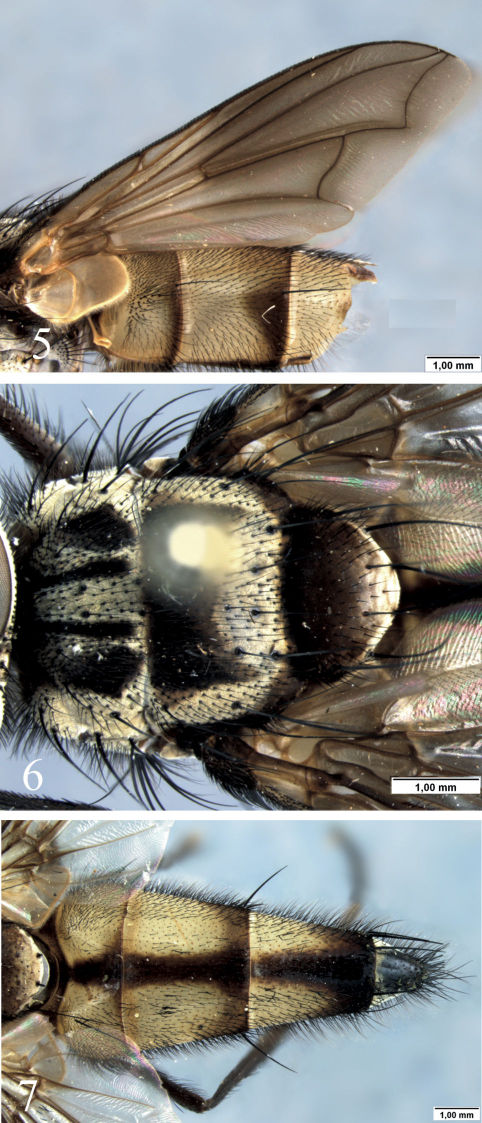
Diaugia angusta: **5** male wing, lateral view **6** male thorax, dorsal view **7** male abdomen, dorsal view. (Scale bar: 1 mm)

(Figures 1–7): Frontal vitta dark-brown to black (Figure 3); head silver or light-golden pruinose but gena with reddish dark brown area from the eye lower margin to vibrissal angle (Figures 1). Antenna dark-brown. Palpus yellowish brown; proboscis dark-brown. Thorax dark-brown to black with silver or light-golden pruinosity (Figure 6); the scutum with 4 dark stripes not reaching the scutellum, and the remaining scutum silver pruinose. Wing hyaline, the membrane tinged with light-brown or at least along the veins (Figure 5); calypteres and halter brown. Legs dark brown with silver pruinosity on coxae and femora. Abdomen (Figures 5 and 7) dark brown to black with silver pruinosity on anterior margin of tergites 3 to 5, and with extensive yellow areas on sides of syntergite 1+2 and tergites 3 and 4, both dorsally and ventrally.

####### Head

(Figures 1, 3): Eye apparently bare, with very short and sparse setulae. About 14 pairs of frontal setae. Two minute proclinate fronto-orbital setulae; fronto-orbital plate bare and narrower than frontal vitta and parafacial. Parafacial bare. Facial ridge with few weak setulae near vibrissa. Postpedicel slender, 4x the length of pedicel; arista long plumose, with 2–3 dorsal and 2 ventral rows (but one single ventral row on basal half), with about same length of postpedicel, and the length of longest cilia about 5x the basal width of arista. Vibrissa fine and long, inserted at level of lower facial margin. Genal dilation covered by fine pale setulae. Palpus filiform; labella developed, slightly shorter than prementum, which is as long as palpus.

####### Thorax

(Figure 6): Acrostichals 3+1, but a weak pair before the postsutural pair. Dorsocentrals 3+4, but seems 3+3 as the second postsutural is weakly developed or indistinct from ground setulae in some specimens. Posthumerals 2, aligned with the intralar row, the posterior seta stronger and the anterior located lateral to the postpronotum. Presuturals 2, the posterior stronger. Postpronotals 3. Notopleurals 2. Postutural intra-alars 2, the anterior weak; intra-postalar absent. Postsutural supra-alars 3, the anteriormost (prealar) weakly developed, about 1/3 the length of the strongest supra-alar and shorter than the first postsutural intra-alar and dorsocentral. Prosternum and proepisternum bare. Six strong anepisternal setae. Katepisternals 2, but some specimens with a reduced lower anterior seta is present. Katepimeron (barette) setulose anteriorly. Scutellum with one basal, one lateral, one apical and one discal pairs of setae.

####### Wing

(Figure 5): costal spine undeveloped; base of R4+5 setulose dorsally and ventrally; M vein bent forward to R4+5, and convex after bend.

####### Legs:

Fore tibia with 2 posterior setae. Mid femur with 2 anterior setae on median third, 2 dorsal preapical setae, and one posterodorsal preapical setae. Mid tibia with 1 submedian anterodorsal seta, 2 posterior setae on apical and basal third (the basal seta weak), and one ventral seta on apical third. Hind tibia with an anterodorsal row of irregularly sized and spaced setae but one strong submedian seta; with one submedian anteroventral and two posterodorsal seta (the submedian stronger).

####### Abdomen

(Figure 7): Abdomen elongate and tapering to apex in dorsal view. Syntergite 1+2 and tergite 3 each with one lateral marginal seta. Tergite 4 with a marginal row of setae, the ventral setae reduced. Tergite 5 with marginal row of setae, with the ventral setae reduced; no discal setae but the ground setulae rather developed dorsally.

####### Terminalia

**Figures 8–10. F3:**
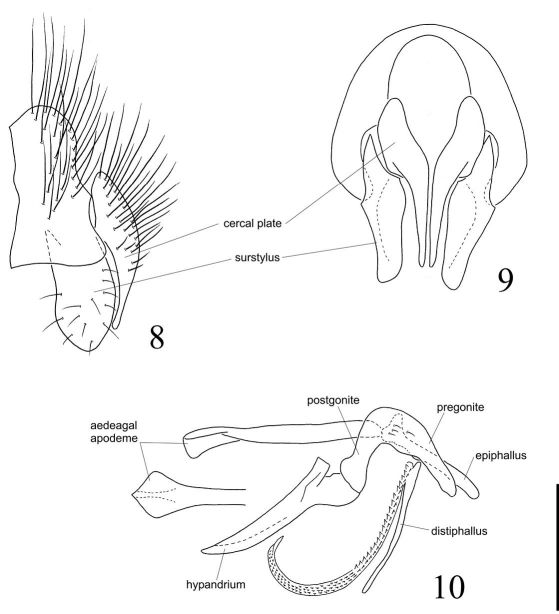
Diaugia angusta: **8** male terminalia, lateral view **9** male terminalia, posterior view **10** aedeagus in lateral view and the detail showing the tip of aedeagal apodeme in dorsal view. (Scale bar: 0.5 mm)

(Figures 8–10): Cercal plate enlarged at base and tapering to the apex in posterior view (Figure 9), with long setulae mostly on base, the tip long and narrow and slightly curved inwards, ending before apex of surstylus (Figure 8). Surstylus broad and the apex rounded, with subparallel margins in lateral view (Figure 9), while in posterior view tapering slightly to the subtruncate apex, the inner surface of surstylus concave. Pregonite and postgonite widely fused as a single piece on each side and firmly connected to the hypandrium (Figure 10); pregonite+postgonite somewhat stout at basal 2/3, strongly curved downwards at middle and tapering to the apex. Aedeagal apodeme straight, with subparallel margins, and elongate, longer than hypandrium (Figure 10). Epiphallus enlarged at base but uniformly narrow until a subtruncate apex, a little longer than half length of aedeagal apodeme. Distiphallus composed of a dorsal sclerite, long, straight and quite narrow, and anteriorly to it, a ventral membrane narrow and extremely elongate. The latter curved at apical half and bearing spinulae all along, these spinulae with their points upwardly oriented and becoming tinier towards the apex (Figure 10).

###### Female:

Body length: 11.0 mm (11.5–15.0), wing length: 9.0 mm (7.5–10.5) (n=12). Differs from male in the following: two strong proclinate fronto-orbital setae (Figures 2, 4); fronto-orbital plate as wide as both frontal vitta and parafacial; fore tibia with 1 posterior seta; abdomen oblong in dorsal view, not elongate as in males, with the yellow areas on sides of syntergite 1+2 and tergites 3 and 4 less extensive than in male; tergite 3 with a median marginal pair; and tergite 5 with the ground setulae weakly developed.

##### Intraspecific variation.

Within the examined material, the specimens showed some significant intraspecific variation in colouration: body colouration varied from dark brown to black (compare Figures 3 and 4); head and thorax with silver to light-golden pruinosity (compare Figures 1 and 2, and 3 and 4); wing always hyaline but the membrane tinged throughout with light-brown (Figure 5) or infuscated at least along the veins, and in some specimens with no noticeable infuscation.

##### Examined material.

BRAZIL: *Goiás*, Anápolis, 1 male, 3.iii.1937, 1 male, vii.1934, Serviço Febre Amarela M.E.S. leg. (MZSP); *Rio de Janeiro*: Itatiaia, 700m, 1 male, 28.iv.1941, J. F. Zikán leg. (MZSP); Rio de Janeiro, Corcovado (Paineiras), 2 males (MZSP), 1 male (MNRJ), iii.1934, L.T. [Travassos] leg. (MZSP); idem, 1 male, xi.1935, L. Travassos leg. (MNRJ); idem, 1 male, 19.i.1938, Oiticica leg. (MZSP); Rio de Janeiro, Jardim Botânico, 1 male, vi.1935[? year hardly readable], H. S. Lopes leg. (MZSP); *São Paulo*: Barueri, 1 male, 3.iv.1957, 1 male, 20.iv.1957, 1 male, 20.vi.1957, 1male, 28.xii.1965, 2 males, 15.i.1966, 1 male, vii.1966, K. Lenko leg. (MZSP); Juquiá, 3 males and 3 females, i.2006, R. Pavarini leg. (ex. Metamasius sp. in “pupunha” crop [Bactris gasipaes Kunth]) (MZSP); Pariquera-Açu, 8 males and 11 females, iv-xii.2007, P.H. Silva & R. Pavarini leg. (ex. Metamasius hemipterus in “pupunha” crop – Bactris gasipaes) (MZSP); Araçatuba, Sítio Santo Amaro, 1 male, 6.i.1963, Rabello leg. (MZSP); Salesópolis, Boracéia, 1 male, 10–14.xi.1947, L. Trav. F., G. Ramalho & E. Rabello leg. (MZSP); idem, 2 males, 14.viii.1947, E. Rabello, Trav. F. & J. Lane leg. (MZSP); São Paulo, Ipiranga, 1 male, i.1932, R. Spitz leg. (MZSP); *Paraná*, Rio Negro, 1 male, 7.i.1929, no collector (MZSP); *Santa Catarina*: Blumenau, 1 male, xii.1924, Luederwaldt leg. (ex. Metamasius ensirostris) (MZSP); Nova Teutônia, 1 male, iv.1964, 1 male, viii.1967, F. Plaumann leg. (MZSP).

##### On the type material.

Perty (1833: 187) described Diaugia angusta without mentioning the composition of the type-series and with no reference to a holotype. Townsend (1939: 77) provided a diagnosis of Diaugia in his “Manual of Myiology” and deliberately referred to a ‘holotype’ and a ‘paratype’ (deposited at “Munich” and “Washington” respectively). Quite possibly he had not examined the supposed ‘holotype’ (perhaps only the ‘paratype’ at the USNM), as there is no clear statement in the brief pages about Diaugia. The male syntype deposited at the Zoologischen Staatssammlung München is lost (Reiss and Schacht 1983: 308), and the only type-material remaining is the male syntype at USNM.

##### Distribution.

**Figure 11.**
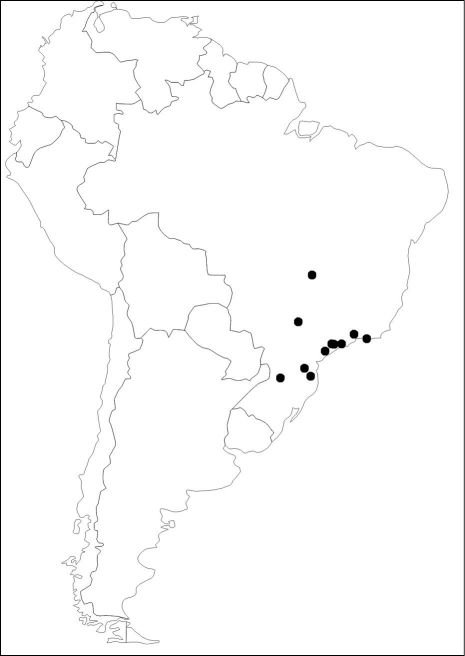
Distribution map of Diaugia angusta (black dots).

BRAZIL (states of Goiás, Minas Gerais, Rio de Janeiro, São Paulo, Paraná, Santa Catarina) (Figure 11).

##### Habitat characterization.

Based on geographical data of the examined material, Diaugia angusta has been recorded in areas covered by Atlantic Forest (Figure 11). Most areas are characterized by dense evergreen ombrophilous tropical forests, whereas there is a single record in semi-deciduous forest (Brazil: São Paulo: Araçatuba). Outside the Atlantic Forest, this species also occurs in drier biomes such as Cerrado (Brazil: Goiás: Anápólis), however, it is very likely that it actually inhabits the gallery forests (humid forests accompanying riverine systems). Additionally, based on the known records, the altitude ranges from sea-level to 900 meters.

##### Hosts:

i) Metamasius ensirostris (Germar, 1824) (Coleoptera: Dryophthoridae) (new record) from Blumenau, state of Santa Catarina;

ii) Metamasius hemipterus (Linnaeus, 1758) (new record) from Pariquera-Açu, state of São Paulo;

iii) Metamasius sp. (new record) from Juquiá, state of São Paulo.

## Preliminary field research

The junior author is developing a long-term research project searching for natural enemies of boring weevils attacking peach-palm crops (“pupunha”, Bactris gasipaes). In a non-commercial crop of peach-palms in the municipality of Pariquera-Açu (southernmost of state of São Paulo), larvae of Metamasius sp. (undetermined species) were observed attacking the stems. Population peaks of the weevil were recorded during the months with higher temperatures and higher levels of precipitation.

Between April and December^ 2007^, a survey was conducted to identify parasitoids of Metamasius sp. in that area. A total of 235 pupae were collected in the field and taken to the laboratory (UNESP, Registro-SP), where they were stored individually in small glass vials covered with a polyester-netting cloth. They were maintained under ambient-temperature until the emergence of the host or parasitoid.

**Figure 12.**
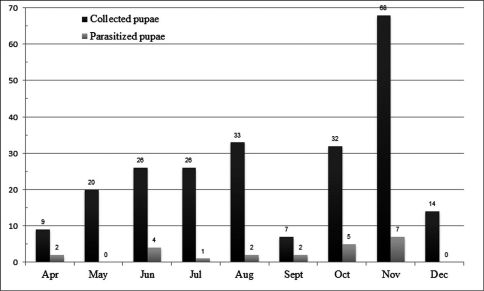
Monthly parasitism of Metamasius sp. by Diaugia angusta (total number of collected pupae and total number of parasitized pupae) from April to December^ 2007^ in Pariquera-Açú (Brazil).

**Figure 13.**
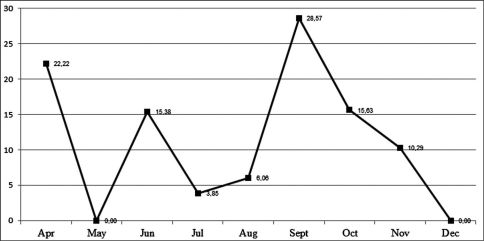
Monthly percentages of parasitism of Diaugia angusta on pupae of Metamasius sp. from April to December^ 2007^ in Pariquera-Açú (Brazil).

Within the 235 field-collected pupae, 23 were parasitized by the tachinid Diaugia angusta. This parasitism varied along the time (Figure 12), ranging from 28.57% in September to 0.00% in May and December (Figure 13). Although the samples were higher in May (20 pupae) and December (14 pupae) than in September (9 pupae), there was no tachinid emergence in May and December. The largest sample was obtained in November with 68 pupae, but only 7 parasitized pupae (10.28%).

We do not have data available for January, February and March, which are usually the months with the highest levels of precipitation and temperature in the study area. Furthermore, additional field studies are required to obtain reliable and statistically significant data on this interaction. Nevertheless, this preliminary field research indicates that Diaugia angusta has a great potential for use in the biological control of Metamasius species in peach-palm crops and, perhaps, in other crops.

## Discussion

**Parasitoidism of tachinid flies on Dryophthoridae and Curculionidae bromeliad and palm pests**

The present study reveals important reports of Diaugia angusta parasitizing Metamasius ensirostris and Metamasius hemipterus.These are the first host records for this tachinid species, and absolutely nothing is known about a potential host specialization over Metamasius ensirostris and/or Metamasius hemipterus. The geographical distribution known for Diaugia angusta ranges from central to southeastern and southern Brazil (Figure 11), while the host Metamasius ensirostris occurs from northern South America (Colombia, Venezuela) southwards to Paraguay, southern Brazil and northern Argentina (Vaurie 1966). And the host Metamasius hemipterus is the most widespread among all the species of Metamasius (Vaurie 1966), occurring in Central and South America (from Mexico to Argentina), with recent introductions reported to other regions (*e.g.*, western Africa, UK, USA, Australia, Philippines) (Vaurie 1966, CABI 2007).

**Figure 14.**
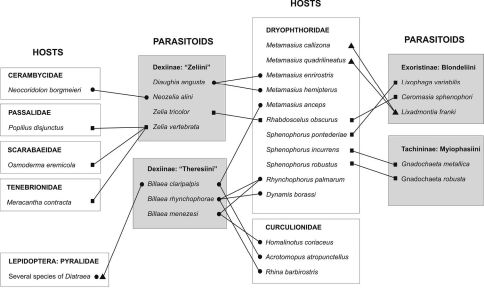
Host-Parasitoid network for Tachinidae, Dryophthoridae and other taxa. Legends: grey boxes = parasitoid taxa; white boxes = host taxa; circles = records in South America; squares = records in North America; triangles = records in Central America. (Sources: Alvarez-Del-Hierro and Cave 1999, Arnaud 1978, Cave 1997, Couturier et al. 1998, Guimarães 1975, 1977, Moura et al. 1993, 2006, Suazo et al. 2006, Wood and Cave 2006).

The host-parasitoid network presented in Figure 14 is focused on the interaction between Tachinidae parasitoids and Dryophthoridae and Curculionidae species reported as pest of bromeliads and palms in the Americas. The network is not complete for every species included, as only “Zeliini” has all its hosts and only Dryophthoridae and Curculionidae have all their parasitoids depicted (see Material and Methods for details).

The network (Figure 14) shows that the parasitoid-host relationship is not very well established in terms of species-specific associations. Dryophthoridae species are parasitized by a great variety of tachinids, and these tachinid species develop on several hosts, not only Dryophthoridae. There are only 10 tachinid species connected to 11 dryophthorid species, on the other hand, these 10 tachinids have several other connections (most not shown) to species of Coleoptera and Lepidoptera (Guimarães 1977, Arnaud 1978). The species of “Zeliini” in the network connect to Cerambycidae, Passalidae, Scarabaeidae and Tenebrionidae (Coleoptera); “Theresiini” connects to Curculionidae (Coleoptera) and Pyralidae (Lepidoptera); Blondeliini connects to Dryophthoridae, Curculionidae, Cerambycidae, Bostrichidae (Coleoptera), Pyralidae, Arctiidae, Hesperiidae, Noctuidae, Olethreutidae, Gelechiidae, Limacodidae, Tortricidae and Noctuidae (Lepidoptera); while Myiophasiini connects to Scarabaeidae and Curculionidae (Coleoptera).

The present study records Diaugia angusta to Metamasius ensirostris and Metamasius hemipterus in southeastern and southern Brazil. Before that, the only host record for a South American “Zeliini” was the one reporting Neozelia alini Guimarães, 1975 on a cerambycid species in southeastern Brazil (Guimarães 1975). Additionally, in North America, Zelia tricolor (Coquillett) parasitizes Rhabdoscelus obscurus (Boisduval) (Dryophthoridae). And, contrary to the apparent specificity observed in “Zeliini”, Zelia vertebrata (Say) has host species within Passalidae, Scarabaeidae, Tenebrionidae and Cerambycidae (Arnaud 1978) (Figure 14).

Members of “Zeliini” has been recorded on several different and not closely related branches of Coleoptera. On this sense, the Dryophthoridae species are also parasitized by a disparity of tachinid species, belonging to three different subfamilies: Exoristinae (Blondeliini), Tachininae (Myiophasiini) and Dexiinae (Dexiini, “Zeliini”, “Theresiini”) (Guimarães 1977, Moura et al. 2006) (Figure 14).

Although the interactions between Dryophthoridae species and the tachinids Lixophaga Townsend, Ceromasia Rondani and Gnadochaeta Macquart seem to be specific, this is an artefact as the network does not show all the host associations recorded to these tachinid genera. North American species of Gnadochaeta parasitize Sphenophorus spp. (Dryophthoridae), but there are also records to several other host species among Curculionidae (Coleoptera), and among lepidopterans Arctiidae, Pyralidae and Noctuidae (Arnaud 1978). Moreover, other Myiophasiini parasitize members of Scarabaeidae and Curculionidae (Coleoptera) (Guimarães 1977). On its turn, Lixophaga is one of the most diverse genus of Blondeliini in the Americas, with 16 Nearctic and 34 Neotropical species, and the host diversity is also high: Dryophthoridae, Curculionidae, Cerambycidae and Bostrichidae (Coleoptera), and Pyralidae, Arctiidae, Hesperiidae, Noctuidae, Olethreutidae, Gelechiidae, Limacodidae and Tortricidae (Lepidoptera).

However, unlike other Blondeliini, Lixadmontia franki Wood and Cave, 2006 (Tachinidae: Exoristinae: Blondeliini) has some specificity in attacking only species of Metamasius. This tachinid has been considered a potential biological control agent of two species of meristem-boring weevils of bromeliads: Metamasius callizona (Cave 1997, Wood and Cave 2006) and Metamasius quadrilineatus Champion (Alvarez-Del-Hierro and Cave 1999). The later species was reported as being parasitized by Lixadmontia franki only during the larval instars IV, V and VI (Alvarez-Del-Hierro and Cave 1999). As far, the available biological information indicates that there is some specificity in the association between Lixadmontia franki and Metamasius callizona and Metamasius quadrilineatus.

## Conclusion

Great efforts have been done recently in order to find parasitoids of bromeliad and palm weevils, by searching for parasitoids for the biological control of Metamasius callizona in Florida (Cave et al. 2003), or by testing the efficiency of parasitoids for the control of Rhynchophorus palmarum in tropical America (Moura et al. 2006).

Although still premature, the use of the tachinid Diaugia angusta as an alternative for the biological control of Metamasius species may be promising, either in its native geographical range or in Central America and tropical North America. On this respect, further studies are needed to test the efficiency of this species under laboratory and natural conditions (through mass hearing and field release). We encourage the community to focus their research on “Zeliini” and “Theresiini” flies while aiming to search for parasitoids of Dryophthoridae and Curculionidae bromeliad and palm pests. Unfortunately, the host records and present knowledge for these tachinids are scattered in the literature. The neotropical “Zeliini” comprises about eleven genera and 18 species, but only four species have some knowledge about their host associations. As for the neotropical “Theresiini”, there are about 14 genera and 23 species, but host records are available to only three species. At present, both “Zeliini” and “Theresiini” are included into the large tribe Dexiini.

## Supplementary Material

XML Treatment for Diaugia
